# Long-term clinical outcomes of the Biomet M2a-38: a retrospective review of 335 total hip arthroplasty cases

**DOI:** 10.1186/s13018-023-04223-4

**Published:** 2023-09-23

**Authors:** Caché S. Jones, Dani Gaillard-Campbell, Thomas P. Gross

**Affiliations:** 1 Midlands Orthopaedics and Neurosurgery, 1910 Blanding Street, Columbia, SC 29201 USA; 2 University of South Carolina, Columbia, SC 29208 USA

## Abstract

**Background:**

At the turn of the century, over one-third of total hip arthroplasties comprised metal-on-metal bearings. As this patient population and their implants age, it is crucial to understand associated late failure modes and expected long-term functional outcomes. We report the long-term results of a large metal-on-metal uncemented total hip arthroplasty system with unique design characteristics compared to others that have been reported with high failure rates.

**Methods:**

We retrospectively analyze our prospective clinical database to determine overall implant survivorship and functional outcomes. Further, we compare these results to the clinical outcomes reported in orthopedic registries and in other published studies with similar metal-on-metal total hip arthroplasty cohorts.

**Results:**

Implant survivorship at 10 years was 99.1% and continued to 97.6% survivorship at 20 years. Implant survivorship at 20 years did not vary significantly between sexes (Male: 98.3%, Female: 97.2%; log-rank *p*-value = 0.46). Mean whole blood cobalt levels were 2.6 µg/L in unilateral cases, 5.3 µg/L in bilateral patients, and 3.4 µg/L for the combined cohort. Average blood chromium levels were 1.4 µg/L in unilateral patients, 2.9 µg/L in bilateral patients, and 1.8 µg/L for group combined. We observed a 0.9% rate of failure due trunnion corrosion at a mean of 13.1 years postoperatively (10.6–15.6 years) but had no bearing wear failures.

**Conclusions:**

Our 20-year implant survivorship of 97.6% with the M2a-38 bearing surpassed registry benchmarks for THA. This large-bearing (38 mm), full hemisphere coverage metal-on-metal system had no bearing wear failures, one failure of instability, one failure of fixation, and three trunnion failures, perhaps suggesting an optimum balance between stability of the joint and the trunnion.

## Introduction

In the mid-2000s, almost 35% of total hip arthroplasty (THA) systems were metal-on-metal (MoM) [1]. As these patient populations and their implant systems age, understanding associated late failure modes and long-term clinical outcomes is critical for providing optimal care. Most metal-bearing THAs have been withdrawn from the worldwide market due to higher reported failure rates than for THAs with other bearing types. One such failure mode that remains poorly understood is trunnion corrosion (TC). Many reports do not distinguish between bearing wear and TC failure, making failure analysis difficult. To attempt to understand the primary modes of failure of MoM in the long-term, we investigated a historical series of Biomet M2a-38 THA systems implanted from 2001 until 2004, after which the larger metal-bearing Magnum THA became available. Based on Australian registry data [2], the Australian Therapeutic Goods Association identified higher than expected revision rates from the Biomet M2a components and issued a hazard alert, with the last known M2a-38 device implanted by another surgeon in 2011 [3].

Although the metal-bearing M2a-38 design is no longer available, it is imperative to maintain routine clinical surveillance of MoM THA patients to understand common failure modes among this substantial patient population and optimize treatment. Going forward, this retrospective review of 335 MoM THAs may be instructive to understand how design features may drive different failure modes. This might help in developing improved implants in the future. Therefore, we now report long-term outcomes with this device.

## Patients and methods

We retrospectively identified 335 THA cases with the M2a-38 THA (Biomet, Inc., Warsaw, IN, USA) performed by a single surgeon (TPG) between November 2001 and November 2004. All postoperative data was collected prospectively. Table 1 presents the demographic information for this study cohort.

**Table 1:** Demographics

Variable	Result
Date range	11/2001–11/2004
# of Cases	335
# Deceased*	160 (47.8%)
*Demographics*	–
% Female	201 (60.0%)
Mean follow-up (years)	9.6 ± 4.9
Age (years)	63.9 ± 14.6
BMI	28.9 ± 6.7
*Diagnoses*	–
Osteoarthritis	259 (77.3%)
Dysplasia	2 (0.6%)
Rheumatoid arthritis	7 (2.1%)
Post-trauma	3 (0.9%)
Legg-calve perthes disease	0 (0.0%)
Slipped capital femoral epiphysis	2 (0.6%)
Osteonecrosis	52 (15.5%)
Other	10 (3.0%)

*Death unrelated to surgery

A posterior surgical approach was used in all cases. Table 2 summarizes surgical data. The M2a-38 acetabular component was high-carbon (> 0.2%) cast cobalt-chrome alloy with a slight peripheral rim flare. The porous coating was standard Biomet titanium alloy plasma spray. The coverage arc on all implant sizes was 180°. Femoral components were either Mallory or Taperloc with the same titanium-plasma spray coating and type 2 tapers without ceramic optimized grooves.

Office or remote follow-up was requested of all patients postoperatively at 6 weeks, 1 and 2 years, and every other year thereafter. A clinical questionnaire, radiographic analysis, and a physical examination testing range-of-motion and strength were performed at each visit; for remote follow-ups, we no longer requested physical examinations after 1-year postoperative. We used our OrthoVault clinical database (Midlands Orthopaedics & Neurosurgery PA, Columbia, SC) for prospective collection and retrospective analysis of demographic, clinical, and radiographic data. We list clinical outcomes in Table 3. We used the information collected from patient questionnaires to calculate the following scores for clinical evaluation: Harris hip score (HHS) for functional assessment [31], University of California, Los Angeles (UCLA) activity score [32], and visual analog scale (VAS) pain score for normal and worst days [33]. UCLA activity scores measure postoperative activity level on a scale from 1 to 10, for which a 10 represents the highest level of activity; VAS pain scores rate the level of pain from 0 to 10, with 0 representing no pain and 10 representing maximum, debilitating pain.

**Table 2:** Surgical data

Variable	Result
Length of incision (in)	3.7 ± 1.4
Operation time (min)	146 ± 107.6
Estimated blood loss (mL)	282.3 ± 175.7
Hospital stay (days)	3.9 ± 1.1
# Transfusion received	0 (0.0%)
Transfusion volume (CC)	–
Acetabular cup size (mm)	54.0 ± 3.6

**Table 3:** Clinical outcomes

Variable	Result (Average)
*Preoperative*	
HHS score	40.4 ± 14.0
*Postoperative*	
HHS score	84.3 ± 14.2
UCLA score	4.2 ± 1.8
VAS pain: regular	0.9 ± 1.9
VAS pain: worse	2.2 ± 3.0

We collected supine and standing anterior–posterior pelvis and lateral radiographs and analyzed these for component position, shifting, and radiolucencies. We determined acetabular inclination angle (AIA) by taking the angle between a measurement line running across the face of the acetabular component and a reference line horizontal across the inferior pubic rami [30]. All measurements were taken using InteleViewer (InteleRAD, Chicago, IL).

Initially, metal ion testing was not part of routine follow-up. Beginning in 2011, we began requesting that all patients obtain metal ion testing at 2-years postoperative. For patients with prior surgeries, we reached out to request testing no matter their postoperative interval. Plasma and serum values were converted to whole blood levels [4].

We performed all statistical analyses using XLSTAT (Addinsoft, New York, NY). Paired, 2-tailed Student’s *t*-tests were carried out to find significant differences between averages. Two-sample proportion *Z*-tests were used to compare ratios between groups. All tests were carried out at *α* = 0.05. Kaplan–Meier (KM) implant survivorship curves were generated using revision as an endpoint to estimate postoperative survival rates of implants. We performed both a log-rank test and a Wilcoxon test to determine whether implant survivorships between groups were statistically different.

## Results

**Fig. 1:**
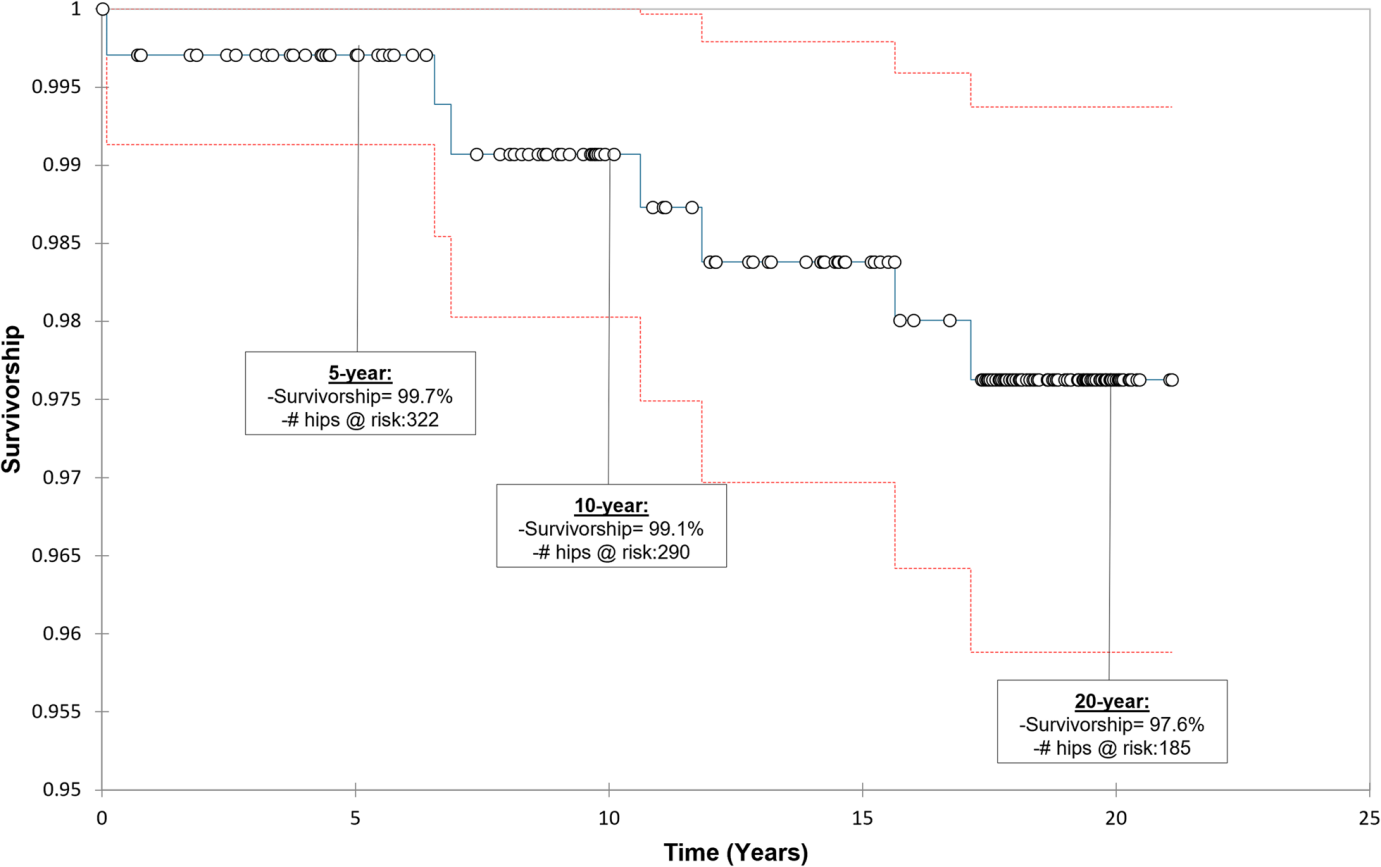
Kaplan–Meier survivorship curve of the M2a-38 total hip implant

For this single-surgeon cohort of 335 M2a-38 Magnum cases, 20-year Kaplan–Meier (KM) implant survivorship was 97.6% (Fig. 1). Implant survivorship at 20-years postoperative did not differ significantly between male and female patients (Male: 98.3%, Female: 97.2%; log-rank *p*-value = 0.46). There were 8 failures (2.4% of cases), including 4 cases of trunnion corrosion and one case each of acetabular cup loosening, femoral fracture, and recurrent instability. There was one case of late hematogenous infection at 17 years postoperative. There were no wear failures. Table 4 summarizes implant failures.

**Table 4:** Failures

Type	Result
# Cases	# (%)
*(1) Acetabular failures*	
Adverse wear	0 (0.0%)
Acetabular component loosening	1 (0.3%)
Failure of acetabular ingrowth	0 (0.0%)
Acetabular component shift	0 (0.0%)
*(2) Femoral failures*	
Trunnion corrosion	4 (1.2%)
Femoral fracture	1 (0.3%)
Femoral component loosening	0 (0.0%)
*(3) Other failures*	
Unexplained pain	0 (0.0%)
Late infection	1 (0.3%)
Recurrent instability	1 (0.3%)
Total failures	**8 (2.4%)**

Bold value indicates the cumulative value

The diagnosis of bearing wear failure was made with the following conditions: whole blood cobalt levels over 15 µg/L, white purulent appearing fluid, a grey lined thick pseudo capsule, and a malpositioned acetabular component (too steep or anteverted). In contrast, a diagnosis of TC was made when the following conditions were met: whole blood cobalt levels over 1.5 µg/L, black debris present on the trunnion with a variable amount of physical damage to the trunnion and/or the head taper itself, dark colored fluid, an inflammatory pseudo capsule, and a well-positioned cup.

All four M2a-38 wear/corrosion failures in this series appeared to be trunnion corrosion cases according to our descriptions above. They all had extensive debridement. None had significant abductor damage. In one case, the head and cup were revised to dual mobility implants using a titanium sleeve with a 28-mm Biolox ceramic head. In two cases, the head only was revised to a 38-mm dual mobility head/liner utilizing a titanium sleeve and a 28-mm Biolox head and the 38-mm M2a acetabular component was retained. In the final case that presented with a dissociated femoral head, the femoral trunnion was so damaged that the femoral component could not be retained. In these cases, both components were revised to a fixed bearing 40-mm ceramic/polyethylene uncemented system.

There were four complications that did not require revision surgery, including 2 cases of unexplained pain (0.6% of total cohort), one case of sciatic nerve palsy (0.3%), and one late trochanteric fracture (0.3%) that was resolved with conservative treatment (Table 5).

**Table 5:** Complications

Type	Result
# Cases	# (%)
Unexplained Pain	2 (0.6%)
Sciatic Nerve Palsy	1 (0.3%)
Fracture	1 (0.3%)
Total complications	**4 (1.2%)**

Bold value indicates the cumulative value

Mean postoperative HHS were 85.6, and mean VAS pain levels were 0.9 on regular days and 2.2 on worst days. Patients were allowed to resume unrestricted activity at 6 months postoperative. Activity level was measured using UCLA activity scores; mean UCLA score at the 2-year postoperative follow-up was 4.2.

Metal ion tests were obtained from 45.1% of patients at an average of 10 years postoperative (Table 6). Plasma and serum metal ions were converted to whole blood values in 40 cases (34.2% of metal ion test results). Mean whole blood cobalt was 2.6 µg/L in unilateral cases and 5.3 µg/L in bilateral patients. Mean whole blood chromium levels were 1.4 µg/L and 2.9 µg/L in unilateral and bilateral cases, respectively. Metal ion values were within “normal” range for 57.3% of all study patients (“normal” is defined as expected metal ion levels for a person without any metal implants, 1.5 µg/L), and they were optimal in 99.1% of all cases (optimal for isolated bearing systems as established by DeSmet) [5]. Besides the cases of trunnion corrosion, no patients had problematic or toxic ion levels. The four trunnion corrosion cases had average cobalt and chromium levels of 11.7 µg/L (range: 6.4–23.5 µg/L) and 9.6 µg/L (range: 2.6–22.6 µg/L), respectively.

**Table 6:** Whole blood metal ion test results

Variables	Unilateral (*N* = 257)	Bilateral (*N* = 78)	*p*-value
Cobalt (µg/L)	2.6 ± 2.3	5.3 ± 3.4	< 0.0001*
Chromium (µg/L)	1.4 ± 1.1	2.9 ± 2.0	< 0.0001*
#, % Patients Tested	151 (45.1%)		–
Normal (#, %)	55 (66.3%)	12 (35.3%)	0.002*
Optimal (#, %)	82 (98.8%)	34 (100%)	0.52
Acceptable (#, %)	1 (1.2%)	0 (0.0%)	0.52
Problematic (#, %)	0 (0.0%)	0 (0.0%)	1.000
Toxic (#, %)	0 (0.0%)	0 (0.0%)	1.000

*Bold *p*-value represents statistical significance

## Discussion

In this study of 335 Biomet M2a-38 metal bearing THA cases, KM implant survivorship at 10 years (99.1%) and 20 years (97.6%) postoperative exceed NICE (National Institute for Clinical Excellence) criteria [6], which defines a 95% 10-year benchmark survivorship for hip arthroplasty. Implant survivorship at 20-years postoperative differed by less than 2% between men and women (*p* = 0.05).

The most prevalent and concerning failure mode within this series was that of TC, occurring in 1.2% of patients. At revision surgery, cold-welding of the trunnion was often observed in these cases and required specialized instruments to remove. In isolated trunnion corrosion cases in metal-on-polyethylene (MoP) THA, we have observed brownish fluid and extensive brown tissue discoloration reminiscent of chronic bleeding. There was occasionally severe damage to the abductor muscles. The cobalt head was easily detached from the titanium trunnion. There was copious black titanium debris on the trunnion and sometimes material loss on the cobalt head. Patient whole blood cobalt levels were mildly elevated (mean 11.7 µg/L, range: 6.4–23.5 µg/L), but not to the extent of bearing wear cases. Neither grey cobalt, nor black titanium were seen in the tissues.

Implant survivorship for metal bearing THA in the registries is lower than for other bearing types. In the 2022 British National Joint Register (NJR) [7], the 15-year KM implant survivorship for uncemented THA implants was 94% for MoP, 78% for MoM and 96% for ceramic-on-polyethylene. In the 2022 Australian Joint Registry [8], 15-year KM implant survivorship for THA was 94% for MoP and ceramic-on-polyethylene, 90% for standard polyethylene, and 73% for large-bearing MoM. Limited data from the Swedish Hip Registry of 2019 [9] indicates a 78% 12-year implant survivorship for large-metal bearing THA. However, registry data is incomplete. The British NJR reports missing data in 3–4% of primary THAs and 5–11% of revision THAs, with another 6% excluded for lack of personal identifier. The Swedish registry reports a reporting frequency of 96% for primary THA and a completeness for revision surgery at 95%. The Australian registry does not provide any data on accuracy of their registry.

We found three other studies reporting on the M2a-38 bearing. Cuckler [10] reported on 616 cases with a lower dislocation risk than 28-mm bearings. However, his follow-up was short and would be too early to evaluate for acetabular fixation failure, trunnion corrosion or excess bearing wear. Lombardi [11] reported a larger series of 636 M2a-38 THA with a 12-year 87% KM survivorship overall. There was a 4% rate of ARMD which represented 48% of failures. The authors did not distinguish between AWRF of the bearing or trunnion corrosion. There was only one dislocation. Minimum 2-year follow-up was achieved on only 85% of cases. 35% of failures were due to failure of socket in growth suggesting a technical problem of these surgeons with implanting this device. Another small series of the M2a-38 bearing in 80 patients with 84% rate of follow-up by Trevisan et al*.* [12] had one failure at 10 years postoperative due to a loose cup and another failure at 13 years postoperative due to an asymptomatic fluid collection with increased blood metal ion levels. KM implant survivorship was 98% at 10 years and 74% by 13 years postoperative. It was not clear if the single ARMD failure was a trunnion corrosion or bearing wear problem or if the trunnion was mismatched. 8 cases had Zimmer stems combined with Biomet bearings which is highly problematic. The mean postoperative cobalt and chromium levels reported by Trevisan et al*.* were comparable to those reported herein.

The Metasul 28-mm MoM articulation has shown excellent outcomes in numerous studies, which have shown implant survivorship from 94 to 98% at 7–15 years postoperative [13–15]. There are few ARMD failures with this implant, which has a full hemisphere cup (180°) and a small head-neck ratio at the mixed metal trunnion connection. Therefore, this implant is not prone to edge loading and AWRF nor trunnion corrosion. It does still suffer from the instability common to small bearing THA.

As is a typical concern with long-term series, limitations of this study mainly arise from incomplete data due loss of follow-up and natural mortality in an elderly population. Nearly 48% of patients were recorded as deceased by the time of this publication, at an average of 18.9 years postoperative. No patients died as a result of their arthroplasty surgery or from complications with their operative hip. We have recorded dates of death for 51% of the deceased cohort. Average time to death was 10.4 years postoperative and 5.6 years after their last completed follow-up. Of the deceased cohort, 100% had a minimum of 2-year follow-up recorded with us. The average HHS for this group at this interval was 93.3 ± 8.3 out of 100. The last recorded follow-up for this group was at a mean of 4.6 years postoperative, where the group presented a mean HHS of 87.0 ± 13.0. From our recorded data and to the best of our knowledge, only one patient from the deceased cohort had an implant revision; this occurred at 6 years postoperative due to recurrent dislocation. Further, our findings are limited in scope by the nature of a single-surgeon series. This makes comparison difficult and could introduce potential bias. However, the primary surgeon does not select against patients on the basis of age, sex, or diagnosis.

## Conclusion

Our reported 97.6% 20-year implant survivorship with 335 uncemented metal-on-metal M2a-38 surpasses registry benchmarks for THA and confounds the widely accepted suggestion that all large-bearing MoM THA systems should be avoided. This study presents the longest follow-up of any large-bearing MoM THA and has a low rate of failure due to acetabular fixation failure (0.3%), instability (0.3%), wear failure (0.0%), and trunnion corrosion (1.2%).

There is historical and ample evidence on the risks of wear failure with MoM bearing systems [16–21]; however, the current results show a lower rate of these failure mechanisms within this M2a-38 series than registry-reported values for MoM THA [2, 7, 9]. Therefore, we suggest a more thorough assessment of bloodwork and radiographic analysis prior to diagnosis to avoid possible unnecessary revision surgery. Due to a current lack of published information on TC, we also encourage detailed documentation of intraoperative findings at revision to distinguish between, characterize, and identify patterns in failure modes.

## Data Availability

Summary data are available in the attached data tables. Deidentified raw data can be found within the Synapse data repository (SynID: syn51671684) or are available upon request.

## References

[1] Bozic KJ, Kurtz S, Lau E, Ong K, Chiu V, Vail TP, Rubash HE, Berry DJ (2009). The epidemiology of bearing surface usage in total hip arthroplasty in the United States. J Bone Joint Surg Am.

[2] Association AO. Australian Orthopaedic Association National Joint Replacement Registry. In. 2021.

[3] Administration ATG. Biomet M2a metal-on-metal total hip replacement implants. In: Administration ATG, (ed). Hazard alert: higher than expected revision rate. https://www.tga.gov.au/news/safety-alerts/biomet-m2a-metal-metal-total-hip-replacement-implants#:~:text=Consumers%20and%20health%20professionals%20are%20advised%20that%20Biomet%2C,these%20components%20have%20higher%20than%20expected%20revision%20rates. 2015.

[4] Smolders JM, Bisseling P, Hol A, van der Straeten C, Schreurs BW, van Susante JL (2011). Metal ion interpretation in resurfacing versus conventional hip arthroplasty and in whole blood versus serum. How should we interpret metal ion data. Hip Int.

[5] Desmet KA, Van Der Straeten C. The interpretation of metal ion levels in unilateral and bilateral hip resurfacing. Clin Orthop Rel Res;2012. 10.1007/s11999-012-2526-x PMC3549185 22930211

[6] Excellence NIfHaC. Total hip replacement and resurfacing arthroplasty for end-stage arthritis of the hip. In: Technology appraisal guidance [TA304]. National Institute for Health and Care Excellence. 2014

[7] Committee NE. British National Joint Registry: 19th Annual Report. 19, 2022 36516281

[8] Aoanjrr. Australian 2018 Joint Replacement Registry. 2018

[9] Paxton EW, Cafri G, Nemes S, Lorimer M, Karrholm J, Malchau H, Graves SE, Namba RS, Rolfson O (2019). An international comparison of THA patients, implants, techniques, and survivorship in Sweden, Australia, and the United States. Acta Orthop.

[10] Cuckler JM, Moore KD, Lombardi AV, McPherson E, Emerson R (2004). Large versus small femoral heads in metal-on-metal total hip arthroplasty. J Arthroplasty.

[11] Lombardi AV, Berend KR, Morris MJ, Adams JB, Sneller MA (2015). Large-diameter metal-on-metal total hip arthroplasty: dislocation infrequent but survivorship poor. Clin Orthop Relat Res.

[12] Trevisan C, Piscitello S, Klumpp R, Mascitti T (2018). Long-term results of the M(2)A-38-mm metal-on-metal articulation. J Orthop Traumatol.

[13] Zuiderbaan HA, Visser D, Sierevelt IN, Penders J, Verhart J, Vergroesen DA (2018). Long-term clinical results of the metasul metal-on-metal total hip arthroplasty: 12.6 years follow-up of 128 primary total hip replacements. Hip Int.

[14] Weber BG (1996). Experience with the metasul total hip bearing system. Clin Orthop.

[15] Delaunay CP, Putman S, Puliero B, Begin M, Migaud H, Bonnomet F (2016). Cementless total hip arthroplasty with metasul bearings provides good results in active young patients: a concise followup. Clin Orthop Relat Res.

[16] Biomet. 10-million cycle wear rates of large metal-on-metal articulations %! 10-million cycle wear rates of large metal-on-metal articulations.

[17] Jack CM, Walter WL, Shimmin AJ, Cashman K, de Steiger RN (2013). Large diameter metal on metal articulations. Comparison of total hip arthroplasty and hip resurfacing arthroplasty. J Arthroplast.

[18] Mantymaki H, Junnila M, Lankinen P, Seppanen M, Vahlberg T, Makela KT (2017). Systematic screening of adverse reactions to metal debris after recap-M2A-magnum metal-on-metal total hip arthroplasty. Scand J Surg.

[19] Law J, Crawford DA, Adams JB, Lombardi AV (2020). Metal-on-Metal total hip revisions: pearls and pitfalls. J Arthroplasty.

[20] Chang J, Haddad F (2020). Revision total hip arthroplasty for metal-on-metal failure. J Clin Orthop Trauma.

[21] van Lingen CP, Ettema HB, Bosker BH, Verheyen C (2022). Ten-year results of a prospective cohort of large-head metal-on-metal total hip arthroplasty : a concise follow-up of a previous report. Bone Jt Open.

